# The evaluation of Animal Bite Treatment Centers in the Philippines from a patient perspective

**DOI:** 10.1371/journal.pone.0200873

**Published:** 2018-07-26

**Authors:** Anna Charinna B. Amparo, Sarah I. Jayme, Maria Concepcion R. Roces, Maria Consorcia L. Quizon, Maria Luisa L. Mercado, Maria Pinky Z. Dela Cruz, Dianne A. Licuan, Ernesto E. S. Villalon, Mario S. Baquilod, Leda M. Hernandez, Louise H. Taylor, Louis H. Nel

**Affiliations:** 1 Global Alliance for Rabies Control, Sta. Rosa City, Philippines; 2 South Asia Field Epidemiology and Technology Network (SAFETYNET), Quezon City, Philippines; 3 Disease Prevention and Control Bureau, Department of Health, Manila, Philippines; 4 Global Alliance for Rabies Control, Manhattan, United States of America; 5 University of Pretoria, Pretoria, South Africa; Wistar Institute, UNITED STATES

## Abstract

**Background:**

The Philippines has built an extensive decentralised network of Animal Bite Treatment Centers (ABTCs) to help bite victims receive timely rabies post-exposure prophylaxis (PEP) at little cost. This study surveyed patients in the community and at ABTCs of three provinces to assess animal bite/scratch incidence, health-seeking behaviour and PEP-related out-of pocket expenses (OOPE).

**Methodology and principal findings:**

During community surveys in 90 barangays (neighbourhoods), 53% of households reported at least one animal bite /scratch injury over the past 3 years, similar across urban and rural barangays. Overall bite/scratch incidences in 2016–17 were 67.3, 41.9 and 48.8 per 1,000 population per year for Nueva Vizcaya, Palawan and Tarlac respectively. Incidences were around 50% higher amongst those under 15 years of age, compared to -those older than 15. Household awareness of the nearest ABTCs was generally over 80%, but only 44.9% sought proper medical treatment and traditional remedies were still frequently used. The proportion of patients seeking PEP was not related to the distance or travel time to the nearest ABTC. For those that did not seek medical treatment, most cited a lack of awareness or insufficient funds and almost a third visited a traditional healer. No deaths from bite/scratch injuries were reported. A cohort of 1,105 patients were interviewed at six ABTCs in early 2017. OOPE varied across the ABTCs, from 5.53 USD to 37.83 USD per patient, primarily dependent on the need to pay for immunization if government supplies had run out. Overall, 78% of patients completed the recommended course, and the main reason for non-completion was a lack of time, followed by insufficient funds. Dog observation data revealed that 85% of patients were not truly exposed to rabies, and education in bite prevention might reduce provoked bites and demand for PEP. An accompanying paper details the ABTC network from the health provider’s perspective.

## Introduction

Rabies is a fatal disease, and patients bitten by animals that may be carrying rabies require prompt access to post-exposure prophylaxis (PEP) [1]. Alongside preventative vaccination of animals, access to PEP is a central tenet of control in rabies endemic countries. Global health organizations are moving towards the recently set goal of an end to human rabies by 2030 [2], and Gavi is considering investing in the procurement of human rabies vaccine for the low income countries that it supports [3]. Data that help determine the optimal allocation of resources between control in human and animal populations will be critical as more countries advance rabies control efforts and seek to reduce human deaths from this zoonotic disease.

Access to PEP has been increasing in some rabies endemic countries, particularly in Asia where the intradermal route of administration has made it more affordable [4], but there has been very little assessment of how well such provision is serving victims of bites from potentially rabid animals.

Over the last decade the Philippines, where rabies remains endemic, has significantly extended its network of Animal Bite Treatment Centers to over 500 across the country. Although the target of 1 ABTC / 100,000 population has not been reached everywhere, poorer provinces have equivalent access to ABTC to wealthier ones [5]. Since 2016, these facilities have been providing free anti-rabies vaccines and subsidized equine rabies immunoglobulin to animal bite/scratch victims. Each ABTC has trained staff and since 2016, a complete course of rabies vaccine has been provided free of charge to patients. Alongside the provision of PEP, national guidelines to vaccinate dogs against rabies are well established throughout the Philippines, although the coverage achieved may not be as high as ideal [6]. Despite these measures, human rabies deaths continue to occur in the Philippines, with an average of 248.7 per year from 2008 to 2016 [5].

However, if treatment at ABTCs remains too difficult, too expensive, or just undesirable for patients to access, the intended prevention of human deaths may still not be realised. The smallest administrative units in the Philippines is a barangay, which could be a village, district or ward and in urban areas may refer to a city neighbourhood. This study used a community survey in 90 barangays (both rural and urban) across 3 provinces to provide data from the patients’ perspective. We estimated the incidence of animal bites, assessed the level of awareness of ABTCs in the community, and the level of their use in the event of a bite incident. We also examined reasons for not using them. The cost per patient to access PEP was also collected from six ABTCs across the same three provinces and patients who failed to complete the course of PEP were interviewed to ascertain the reasons for this.

By examining awareness of where to seek PEP, and the frequency with which communities access it, this study provides data that can be used to determine the best future strategy to minimise human deaths from rabies in the Philippines and elsewhere. This study was carried out in conjunction with a study of the operation of the network from a health providers perspective which collected data from the same study provinces and described the development of the ABTC network across the Philippines, including its costs and impacts [5].

## Methods

The data presented here are tightly linked to that presented in an accompanying paper from the health care provider’s perspective [5] where more information on the ABTC network in the Philippines can be found, and where the choice of study provinces and ABTCs are more fully explained. Briefly, three provinces were selected to reflect a range of different human population densities and geographies most applicable to Gavi-eligible countries in Africa and Asia. They were Nueva Vizcaya, a mountainous and mostly rural province with a human population density of 100 people/km^2^, Palawan, an island archipelago with a human population density of 65/km^2^ and Tarlac, mainly lowland with more urban areas and a human population density of 450/km^2^.

### Community surveys

For each of the three provinces, a total of 30 barangays were selected using cluster sampling, with the probability of their being selected proportional to their population size. Barangays, which are the smallest administrative units in the Philippines, have been considered as the sampling unit in this study because of their clear boundaries and because Filipino social structure is oriented around the barangay and its officials including health workers We used the classification of barangays as rural or urban from the Philippine Standard Geographic Code (PSGC) [7]. Household interviews were conducted between March 19^th^ and May 4^th^ 2017. Household sampling in these barangays was random (where household master list or spot map was available) or systematically started at a randomly assigned house. Subsequent households that were interviewed were those nearest to the preceding household following a randomly chosen direction determined prior to the start of the survey in each barangay. At every house surveyed, a respondent over 16 years old was interviewed about the household size and animal bites or scratches occurring in the past 3 years to allow estimation of incidence. Bites/scratches occurring outside the province were excluded from further analysis. More detailed data on health seeking behaviour was collected in households with bite incidents, and household interviews were continued until a minimum sample size of 18 bite incidents per barangay was reached. Traditional medicine is still widely practiced in the Philippines, and most communities will have a tandok (traditional healer) who uses herbal medicine to treat illnesses. We collected data on how often patients consulted a tandok following bite/scratch injuries.

Travel time to the nearest ABTC was calculated for each barangay surveyed, using Google Maps to estimate the travel time by car to the ABTC from the central point of the barangay. Central points were assigned to, in order of priority: the barangay hall, public elementary school, or the geographical center of the barangay.

### ABTC patient survey

In each province, one ABTC situated in the province capital and one ABTC in a rural municipality were included to assess patient costs in accessing PEP (Table 1, S1 Fig). The Philippines Department of Health supplies an extensive national network of ABTCs with only high quality, imported rabies vaccine and equine Rabies Immunoglobulin (eRIG). Human RIG is not provided. Vaccine is delivered almost exclusively using the intradermal route following the modified 2-site (Thai Red Cross) regimen (4 visits on days 0, 3, 7 and 28, 8 doses, 2-2-2-0-2), and previously immunised patients are given just two booster doses (1 dose on each of 2 visits) [5]. ABTCS are allowed to charge patients for consumables, such as syringes and for eRIG if required, but cannot charge for government provided vaccine. Patients also need to cover their travel expenses and the cost of their time to attend the ABTC.

**Table 1:** Description of study ABTCs.

Province	ABTC	Classification	Location	Established	Average patients treated per month
Nueva Vizcaya	Nueva Vizcaya Provincial Health Office	Urban	Bayombong Municipality, (Provincial Capitol)	2005	200–360 (2012–15) 590 (2016)
	Alfonso Castaneda Rural Health Unit*	Rural	Alfonso Castañeda Municipality, 5 hours travel by land south of Bayombong	2014	10–13 (2014–15) 13 (2016)
Palawan	Ospital ng Palawan	Urban	Puerto Princesa City	1991	100 (2013–15) 140 (2016)
	Southern Palawan Provincial Hospital	Rural	Brooke’s Point Municipality, 4 hours travel by land south of PPC	2010	30–70 (2012–15) 80 (2016)
Tarlac	Tarlac Provincial Health Office	Urban	Tarlac City	1994	400–680 (2012–15) 780 (2016)
	Paniqui General Hospital	Rural	Paniqui Municipality, 30 minutes travel by land from Tarlac City	2016	12 (2016)

* Does not provide eRIG

In each province, a minimum sample size of 355 to 370 patients arriving for their first visit were targeted across the two ABTCS selected and followed for their entire treatment period. Because of the very different numbers of patients treated per month this could not be evenly divided across the rural and urban ABTCs, but generally all patients at rural ABTCs were interviewed. Where there were more patients available for interview, the patients interviewed were divided across the 6-week data collection period. These patients were selected randomly, and as often as possible, were interviewed in adequately spaced intervals to represent those coming in for consultation at different times of the day.

Patients were interviewed between February and April 2017, during their scheduled PEP visits (Days 0, 3, 7, and 28) in the ABTC. Those who did not return for their scheduled dose were followed-up through phone or home visits (where possible). Data about their bite/scratch incident and all costs associated with their wound treatment were collected. These included direct costs (vaccines and other medical supplies needed) and indirect costs (transportation, meals, and salaries lost). Reasons for missing the scheduled visits were collected from those who had not returned. During the Day 28 follow-up, the status of the biting animal (alive, dead, killed, missing/lost, or unknown after 14 days) was also recorded. The 14 day observation period is stipulated by the Philippines National Rabies Committee for the collection of this data.

### Statistical analysis

Regression and ANOVA analyses were carried out in Excel 2013, Professional edition using the Excel add-in Analysis ToolPak.

### Ethics statement

Ethical clearance was granted by the National Ethics Committee of the Philippines Council for Health Research and Development (NEC Code: 2017-008-Taylor-ABTC, Study Title: The Evaluation of Operating Animal Bite Treatment Centers in the Philippines).

Written informed consent was obtained from adults included in the survey, or from parents or guardians if the subject was a minor.

## Results

### Findings from the community surveys

A total of 1,011, 1,395 and 1,131 households were interviewed from 30 barangays of Nueva Vizcaya, Palawan and Tarlac respectively (Table 2). Overall, an average of 33.7, 45 and 37.7 households per barangay were interviewed for Nueva Vizcaya, Palawan and Tarlac respectively, with a minimum of 12 and a maximum of 86 (both in Palawan).

**Table 2:** Household data collected from the community based study.

Province	Barangays	Households interviewed	Average household size	Knew where to go for PEP	Knew that PEP was free	Households (%) with Injuries	Total injuries recorded	Average injuries per household
**Nueva Vizcaya**	**30**	**1011**	**4.73**	**674 (66.7%)**	**570 (56.4%)**	**562 (55.6%)**	**672**	**0.66**
Rural	29	981	4.73	648 (66.1%)	558 (56.9%)	540 (55.0%)	647	0.66
urban	1	30	4.73	26 (86.6%)	12 (40.0%)	22 (73.3%)	25	0.83
**Palawan**	**30**	**1395**	**4.90**	**1160 (83.2%)**	**561 (40.2%)**	**540 (38.7%)**	**606**	**0.43**
rural	21	981	4.77	824 (84.0%)	392 (40.0%)	375 (38.2%)	423	0.43
urban	9	414	5.19	336 (81.2%)	169 (40.8%)	165 (39.9%)	183	0.44
**Tarlac**	**30**	**1131**	**5.09**	**801 (70.8%)**	**501 (44.3%)**	**540 (47.7%)**	**613**	**0.54**
rural	25	921	4.99	626 (68.0%)	434 (47.1%)	450 (48.9%)	511	0.55
urban	5	210	5.53	175 (83.3%)	67 (31.9%)	90 (42.9%)	102	0.49
TOTAL	**90**	**3537**	**4.10**	**2635 (74.5%)**	**1632 (46.1%)**	**1642 (46.4%)**	**1891**	**0.53**

In all urban barangays and in the rural barangays in Palawan, over 80% of respondents were aware of where to seek PEP (Table 2). In rural barangays of Nueva Vizcaya and Tarlac this fell to 66.1% and 68.0% respectively. Of those who knew where to seek PEP, government health workers were a key source of this information (44.4%, 73.5% and 58.6% of respondents in Nueva Vizcaya, Palawan, and Tarlac) together with neighbours and family (56.8%, 20.9% and 40.3%). Less than 5% of respondents in each province had learned this information from television.

Across all barangays 46.1% of respondents said that they would not have to pay for PEP at an ABTC, and this was lower in the urban barangays for Nueva Vizcaya and Tarlac (Table 2).

Overall 1,642 households reported bites or scratches (46.4%) and a total of 1,891 bite/scratch incidents (suffered by 1,830 bite victims) were reported, with a maximum of 5 injuries (suffered by up to 5 different households members) reported per household. No injuries were reported as having resulted in death. The average numbers of bites/scratches reported per household over the whole 3.25 year period recorded were 0.66, 0.43 and 0.54 for Nueva Vizcaya, Palawan and Tarlac respectively with no strong differences between rural and urban barangays noted (Table 2).

Bite/scratch incidence for the years 2014, 2015 and 2016–17 (the latter based on 1.25 years of data up to March 2017) was calculated for each province by dividing the bites recorded during that year by the total household population that year (accounting for family members born, moved in or out and died). It was further disaggregated by age class to compare incidence in the under 15 age category from that in over 15 age category and by rural and urban barangays (Table 3).

**Table 3:** Bite/scratch incidences per 1,000 people per year for each province, by age group and by rural/urban barangays.

Province	2014			2015			2016–17*		
	<15	≥15	Total	<15	≥15	Total	<15	≥15	Total
**Nueva Vizcaya**	37.4	20.4	25.2	43.9	26.1	31.3	86.7	58.3	67.3
**Palawan**	24.2	13.2	17.0	26.8	14.3	18.7	51.2	36.6	41.9
**Tarlac**	39.7	18.1	24.8	36.2	15.0	21.8	65.1	40.6	48.8
	Rural	Urban	Total	Rural	Urban	Total	Rural	Urban	Total
**Nueva Vizcaya**	24.7	41.4	25.2	31.2	34.7	31.3	66.9	78.9	67.3
**Palawan**	17.7	15.5	17.0	17.6	21.1	18.7	43.4	38.7	41.9
**Tarlac**	24.3	26.9	24.8	21.6	22.3	21.8	52.9	32.4	48.8

* (The 2016–17 estimates are based on data from the 1.25 year period up until March 2017, adjusted to an annual incidence)

There was strong evidence of recall bias, with the time period 2016–17 (1.25 years) showing incidences from 1.97 to 2.66 times higher than 2014 for each province (Table 3). Across all 3 provinces, the percentage of these injuries that were scratches rose from 2014 to 2016–17 in all provinces, and across all provinces it rose from 15.0% to 17.0% to 23.5% of injuries (see S1 Table). This suggests that scratches may be more likely to be forgotten than bites injuries over longer time periods.

Bite/scratch incidences were consistently higher for Nueva Vizcaya across the years, with the 2016–17 mean incidence being 67.3 injuries per 1,000 people per year) compared to 48.8 for Tarlac and 41.9 for Palawan. The number of injuries reported for 2016–17 varied significantly across the provinces compared to that expected if all had the same basic incidence (Chi-sq = 45.33, d.f. = 2, p<0.01).

**Fig 1:**
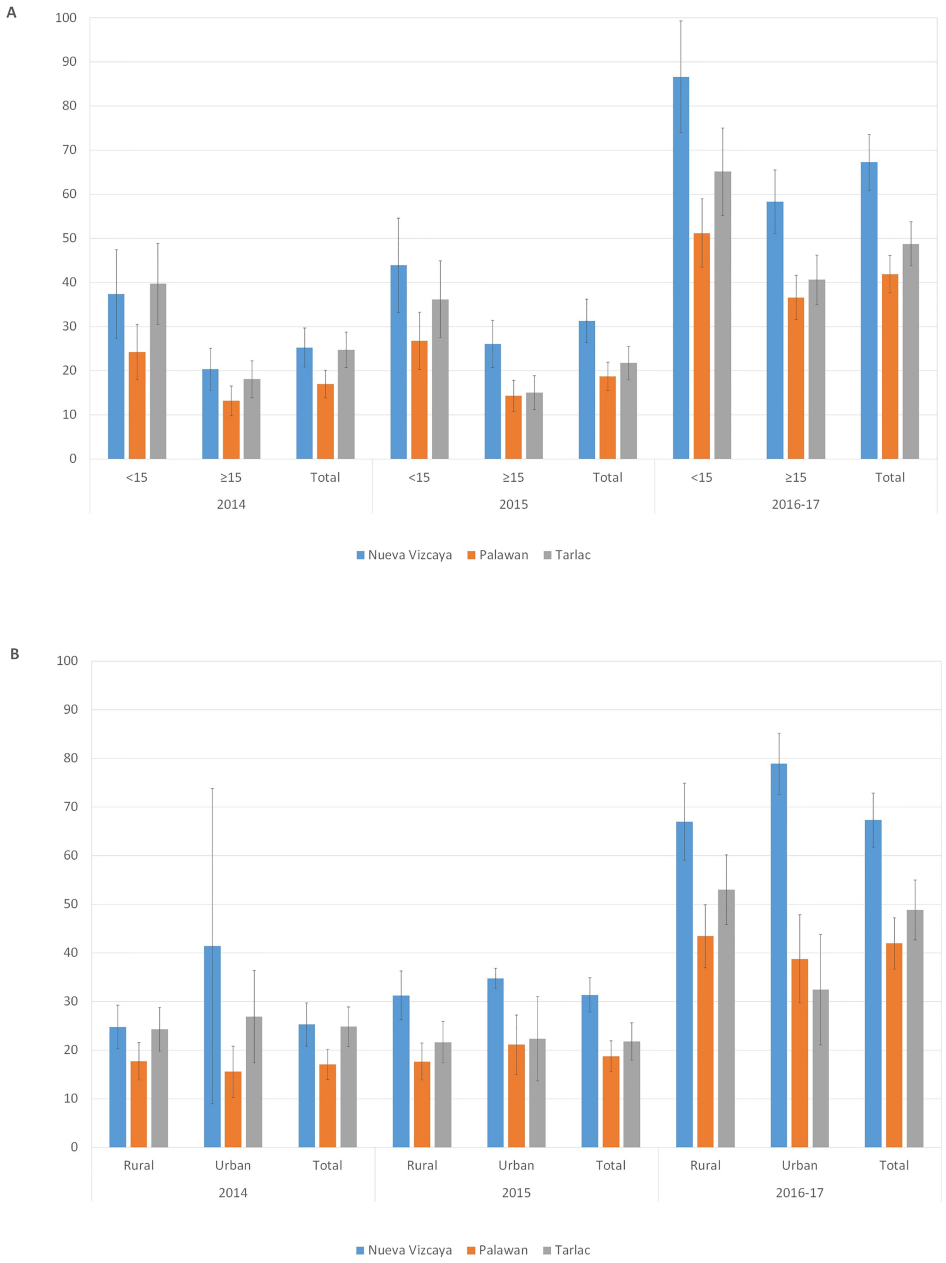
Incidences of animal bites and scratches by year across the three provinces, (A) by age categories and by year (B). The 2016–17 period was 1.25 years, but adjusted to a yearly incidence.

Overall incidences were consistently higher for the under 15 years of age group compared to the over 15 of years group. For 2016–17, the incidence was 1.49, 1.40 and 1.60 times higher in the under 15 years of age category compared to the over 15 years category for Nueva Vizcaya, Palawan and Tarlac respectively (Fig 1A). There was no consistent pattern across rural and urban barangays (Fig 1B).

#### Proportion of bite victims reporting to bite treatment facilities

Given the evidence of recall bias in the data, and the changes in ABTC policy regarding payment for treatment, only the data from 2016 and 17 (total of 1,111 injuries) were included in the following analyses.

Of the 1,111 bites/scratches, most were caused by dogs, but overall almost a third of injuries were caused by cats (Table 4). Most injuries caused by cats (56.9% of 346) were scratches without bite wounds. In contrast 91.9% of the 752 injuries caused by dogs involved bites.

**Table 4:** Characteristics of animal Injuries and their treatment across the three provinces in 2016–17.

	Nueva Vizcaya	Palawan	Tarlac	All provinces
**Number of wounds**	**402**	**358**	**351**	**1111**
Dog inflicted (%)	79.6%	60.3%	61.5%	67.7%
Cat inflicted (%)	19.7%	38.3%	37.0%	31.1%
Bites without scratches (%)	85.6%	62.6%	74.1%	74.5%
Bites with scratches (%)	1.7%	3.1%	1.1%	2.0%
Scratches only (%)	12.7%	34.4%	24.8%	23.5%
**Number of wounds treated in medical facility**	**178**	**181**	**140**	**499**
All wounds treated (%)	44.3%	50.6%	39.9%	44.9%
Bites treated (%)	49.6%	63.4%	45.5%	52.1%
Scratches treated (%)	7.8%	26.0%	23.0%	21.5%
Wounds treated in under 15 age category (%)	50.6%	53.2%	44.9%	49.6%
Wounds treated in over 15 age category (%)	39.9%	48.2%	35.9%	41.3%
Went to ABTC (%)	86.5%	75.7%	87.9%	83.0%
Went to other govn. facility (%)	10.7%	13.8%	8.6%	11.2%
Went to private facility (%)	2.2%	5.5%	3.6%	3.8%
No information (%)	0.6%	5.0%	0.0%	2.0%
Also went to a tandok* (%)	15.2%	0.6%	16.4%	10.2%

*a tandok is a traditional healer

Overall, less than half (44.9%) of victims sought treatment for wounds in a medical facility, but in all provinces a higher percentage of victims sought treatment for bites (average 52.1%) compared to scratches (average 21.5%). Victims seeking treatment did so overwhelmingly at ABTCs (83.0% of all wounds treated) and other government facilities (11.2% of wounds treated), with only 3.8% seeking treatment at private facilities (Table 4). In addition to seeking medical treatment 10.2% of all victims across all provinces also visited a *tandok* (traditional healer), though this was markedly less common in Palawan, where the proportion of bites treated in a medical facility was also higher than the other two provinces (Table 4).

In total, 478 wounds were suffered by those under 15 years, and 632 by those aged 15 years and over. The proportion of wounds treated was slightly higher for the under 15 years group (49.6% across all provinces) compared to the over 15 years group (41.3% across all provinces).

**Fig 2:**
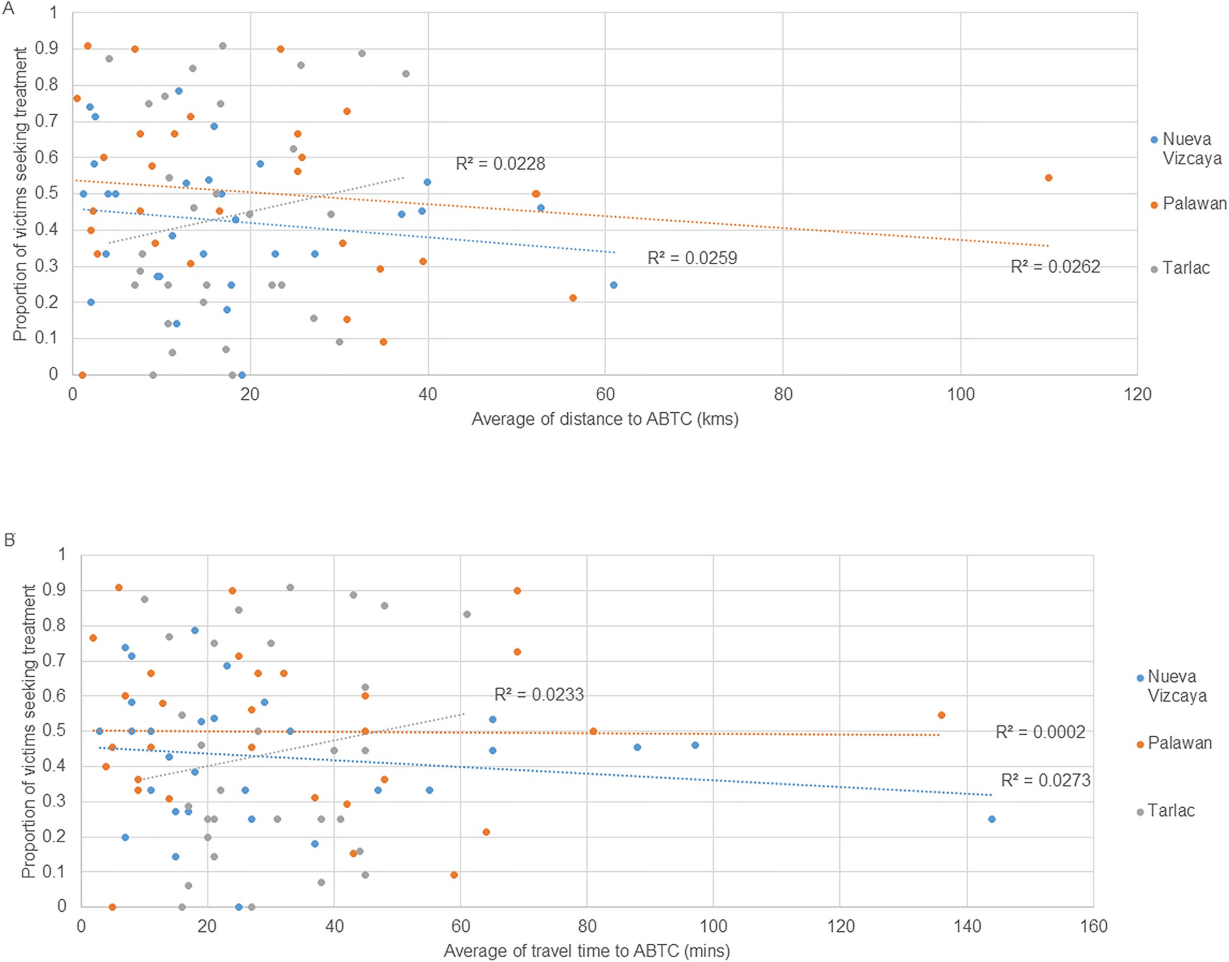
The proportion of victims that sought treatment in 2016 and 2017, related to the barangays distance from (A) and travel time to (B) the ABTC.

Across the 90 barangays sampled, the proportion of victims seeking treatment for wounds in 2016 and 2017 was not influenced by the distance from the barangay to the nearest ABTC, or by the travel time to the nearest ABTC (Fig 2).

#### Wound management practices by those who do not seek medical attention

Data was collected on a total of 612 wounds sustained in 2016–7 where no medical treatment was sought. Overall, 32.7% of victims did nothing at all (not even washing the wound), but there was evidence of a variety of practices, and variations in the number of people practicing them across the provinces (Table 5).

**Table 5:** Wound management practices in 2016–7 by those who did not seek medical treatment (non-exclusive answers).

	Nueva Vizcaya	Palawan	Tarlac	Total
Number of wounds sustained	**224**	**177**	**211**	**612**
did nothing at all	37.9%	18.1%	39.3%	32.7%
visited a *tandok (%)*	37.5%	0.0%	47.4%	30.1%
** Wound washing**				
washed the wound with soap and water	47.8%	63.8%	54.5%	54.7%
washed with water only	0.4%	1.1%	0.0%	0.5%
** Home remedies**				
applied garlic	6.7%	19.2%	3.3%	9.2%
applied papaya	6.3%	1.1%	0.5%	2.8%
applied salt	3.6%	0.0%	1.9%	2.0%
applied ‘rabies tree’ herb	0.0%	2.8%	0.0%	0.8%
applied other herbal medicine	1.3%	10.7%	0.5%	3.8%
applied more than one herbal remedy	2.2%	0.6%	0.9%	1.3%
bled the wound	3.1%	6.2%	3.8%	4.2%
applied alcohol	3.6%	2.3%	0.0%	2.0%
applied antibiotic	0.9%	0.0%	0.5%	0.5%
applied antiseptic	0.0%	0.6%	0.0%	0.2%
applied gasoline and burnt it	0.0%	0.6%	0.0%	0.2%
took unknown medicine	0.9%	0.0%	0.0%	0.3%

Between 47.8% and 63.8% of these victims washed the wound with soap and water, and in Nueva Vizcaya and Tarlac, 37.5% and 47.4% respectively went to a *tandok* (traditional healer). Interestingly, no victims visited a *tandok* in Palawan, despite them being available. A variety of different herbal home remedies were applied, sometimes in combination (see Table 5). In Palawan, but not the other provinces, residents believe that a local plant called the ‘rabies tree’ can prevent rabies, and several respondents reported using this as a home remedy. Between 2.7% and 5.6% bled the wound.

#### What factors prevent patients from accessing bite treatment facilities?

Reasons given for not accessing medical treatment are given in Table 6, with not knowing about the need to seek medical treatment and a lack of money being the primary reasons given. Less than 5% of people overall, and notably none in Palawan listed that they did not know where the ABTC was as the reason for not seeking medical treatment.

**Table 6:** Reasons given for not seeking medical treatment (non-exclusive answers).

	Nueva Vizcaya (n = 224)	Palawan (n = 177)	Tarlac (n = 211)	Total (n = 612)
Didn't know needed to go	37.5%	20.9%	51.2%	37.4%
No money	23.7%	15.3%	28.0%	22.7%
Not a severe wound	16.1%	43.5%	4.3%	19.9%
Too far	11.2%	2.8%	2.8%	5.9%
Belief in tandok	5.8%	2.3%	8.1%	5.6%
Didn't know where ABTC was	8.0%	0.0%	1.9%	3.6%
Busy	7.6%	1.7%	0.5%	3.4%
Other reasons given	10.7%	15.8%	5.7%	10.5%

Details of the other reasons given are included in S2 Table. Interestingly a small number of people said that they did not seek treatment because the dog was vaccinated, that the victim was already vaccinated, or that they chose to observe the dog instead.

### Findings from the ABTC patient cohort

A total of 1,105 patients were interviewed over a 6 week period in early 2017 in the 6 ABTCs, with the vast majority of these patients treated by the urban ABTCs (Table 7). A total of 43 (3.9%) patients across all the sites were lost to follow-up by Day 28.

**Table 7:** Characteristics of patients included in the ABTC survey, 2017.

Characteristics	Nueva Vizcaya		Palawan		Tarlac		Total
	Urban ABTC	Rural ABTC	Urban ABTC	Rural ABTC	Urban ABTC	Rural ABTC	
Total	320	47	235	130	326	47	1105
Sex							
Male	165 (52%)	22 (47%)	132 (56%)	65 (50%)	161 (49%)	24 (51%)	573 (52%)
Female	155 (48%)	25 (53%)	103 (44%)	65 (50%)	165 (51%)	23 (49%)	532 (48%)
Age range (years)							
0 to 5	75 (23%)	10 (21%)	41 (17%)	27 (21%)	86 (26%)	4 (9%)	243 (22%)
6 to 14	67 (21%)	11 (23%)	56 (24%)	27 (21%)	78 (24%)	10 (21%)	249 (23%)
15 to 30	50 (16%)	13 (28%)	53 (23%)	30 (23%)	71 (22%)	9 (19%)	226 (20%)
31 and up	128 (40%)	13 (28%)	85 (36%)	46 (35%)	91 (28%)	24 (51%)	387 (35%)
Biting animal							
Dog	247 (77%)	34 (72%)	184 (78%)	93 (72%)	263 (81%)	36 (77%)	857 (78%)
Cat	72 (23%)	7 (15%)	49 (21%)	36 (28%)	61 (19%)	11 (23%)	236 (21%)
Other^$^	1 (0%)	6 (13%)	2 (1%)	1 (1%)	2 (1%)	0	12 (1%)
Biting animal owner*							
Patient’s family	175 (55%)	21 (51%)	108 (46%)	62 (48%)	169 (52%)	28 (60%)	563 (51%)
Neighbor/ Relative	123 (38%)	20 (49%)	92 (39%)	48 (37%)	137 (42%)	15 (32%)	435 (40%)
Unowned / Unknown	22 (7%)	0	35 (15%)	20 (15%)	20 (6%)	4 (9%)	101 (9%)
Willingness to travel to a different ABTC							
Willing	315 (98%)	46 (98%)	227 (97%)	127 (98%)	227 (70%)	10 (21%)	952 (86%)
Not willing	5 (2%)	1 (2%)	8 (3%)	3 (2%)	99 (30%)	37 (79%)	153 (14%)

^$^ 5 patients were bitten by pigs, 1 was bitten by a monkey and 6 patients in the NV rural ABTC were exposed to a patient with rabies.

*The 6 patients exposed to the human patient are excluded

Overall 52% of all the patients included in the study were male, and 45% of all patients were below 15 years old with some minor variation across ABTCs (Table 7). The majority of the incidents involved dogs and almost all of the biting animals were owned. Willingness to travel to an alternative ABTC was generally high, but lower for the rural ABTC in Tarlac.

#### Patient completion rates

**Fig 3:**
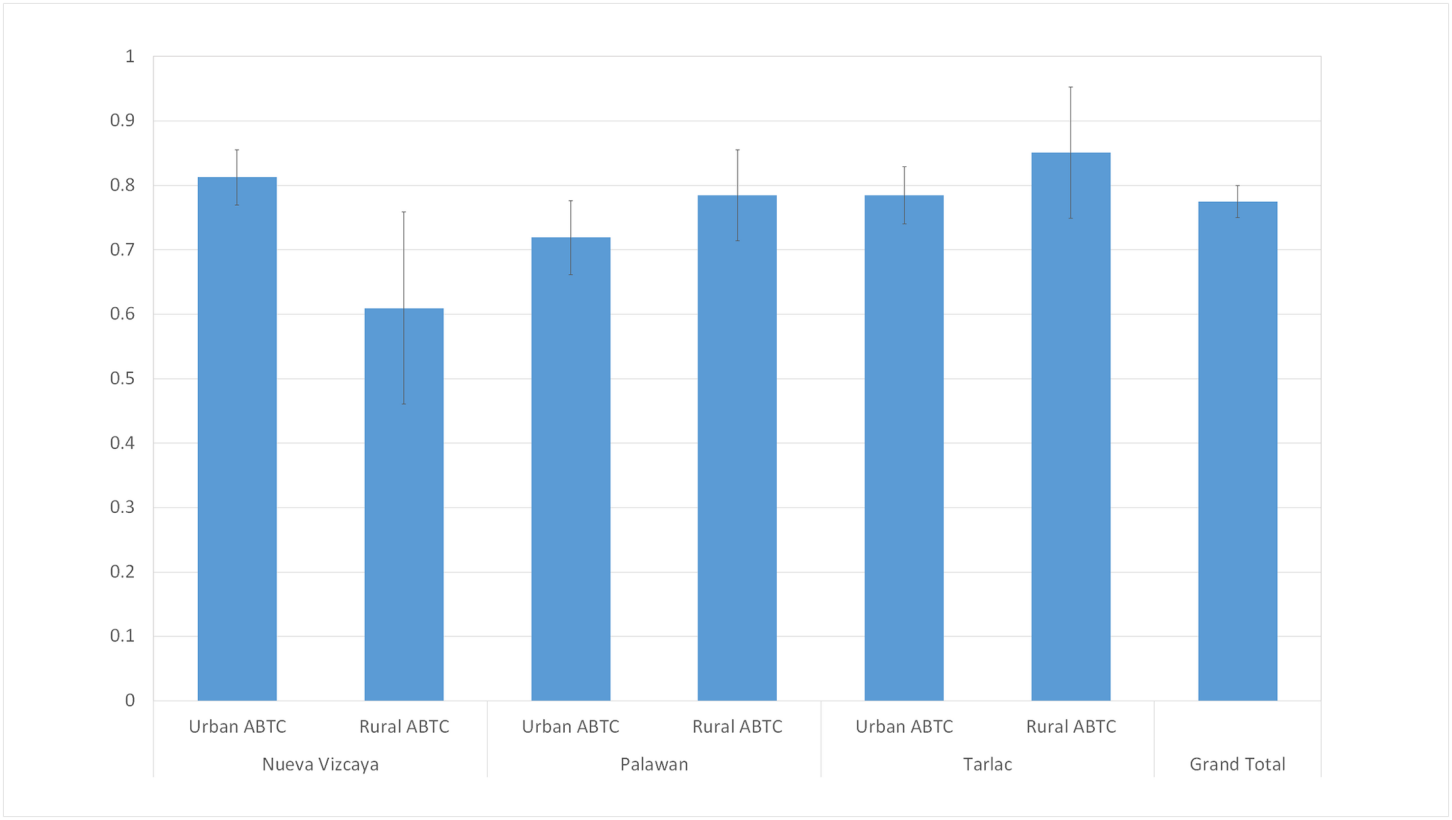
Completion rates of patients requiring PEP in the 6 study ABTCs, 2017.

Overall, 78% of the patients who needed vaccine completed their recommended number of doses. This falls short of the 90% completion rate target set by the national Department of Health (DOH) for 2016. The vast majority (927/1,105) of bite patients were recommended a shortened schedule based on information that the biting animal was still alive after 14 days. The highest observed completion rates were in the Tarlac rural ABTC (85%) and Nueva Vizcaya urban ABTC (81%). The lowest (61%) was observed in the rural ABTC in Nueva Vizcaya. (Fig 3).

The overall completion rates are further disaggregated in Table 8 to compare the average number of visits each patient completed and the number recommended. In general, these figures are consistent with the overall rates per ABTC. Those involved in incidents where the biting animal died however, are more likely to miss visits, particularly on day 28.

**Table 8:** Average number of PEP visits per patient.

Circumstances	Number of patients	Recommended number of visits (doses)	Nueva Vizcaya		Palawan		Tarlac		Total
			Urban ABTC	Rural ABTC	Urban ABTC	Rural ABTC	Urban ABTC	Rural ABTC	
Dog was alive after 14 days	927	3 (6)	2.99	2.47	2.81	3.00	2.80	3.08	2.88
Dog died/unknown status after 14 days	166	4 (8)	3.49	3.25	2.93	3.28	3.40	3.67	3.29
Required booster doses only	12	2 (2)	0.75	--	0.92	--	1.00	--	0.92

#### Reasons for not completing PEP series

Reasons for not returning to the ABTC on their scheduled PEP visits are listed in S3 Table. Reasons differed across all ABTCs but having no time to go back to the ABTC was the most common. Failure to remember the schedule was high (53%) in the rural ABTC in Nueva Vizcaya, which was not observed in the other ABTCs. Lack of funds was cited the most for the rural ABTC in Tarlac (38%). Around a third of the defaulters from both Palawan ABTCs felt that they did not need to return to the ABTC for their Day 28 dose.

#### Status of the biting animal and rabies risks

Of the 1,105 patients interviewed in the 6 ABTCs, 939 (85%) of the biting animals involved were still alive and had not developed rabies after the 14^th^ day, when patients were asked at the day 28 follow-up (S5 Table). Of the 15% incidents involving suspicious biting animals, 66 (40%) died within 14 days while the remaining 100 had unknown statuses. Thus in retrospect we can say that only 9% to 19% of all the patients across the 6 ABTCs, with the highest in the rural ABTC in Palawan, were involved in bite incidents considered high risk (i.e. the dog died).

Of the 166 patients bitten by animals that died or whose status was unknown, 56% received an incomplete course of PEP (S6 Table). Incomplete in this context was defined as less than 8 doses (4 visits). The completion rate was highest in the Tarlac rural ABTC at 83%, while the lowest was in the Palawan urban ABTC (19%).

#### Availability of free vaccine and RIG

Since 2016, the Philippine government has aimed to provide a complete course of vaccine and 1 vial of RIG free to all patients requiring PEP. Table 9 shows the actual number of patients given vaccine or eRIG, and the number who had to pay for at least 1 vial of vaccine, when free stocks had run out in the ABTC. A patient who needed eRIG was defined as any patient classified as Category 3 who tested negative for the skin test. With the exception of Tarlac urban ABTC, almost all of the vaccine given to patients were provided for free by the government. In contrast, the Tarlac urban ABTC had the highest availability of free eRIG. All the other ABTCs were only able to provide 4% to 55% of the total Category 3 patients needing eRIG. 32 patients were prescribed RIG but did not receive it, with the main reason given being not enough money to buy it.

**Table 9:** Required vaccines and out-of-pocket expenses for the patient cohort.

	Nueva Vizcaya		Palawan		Tarlac	
	Urban ABTC	Rural ABTC	Urban ABTC	Rural ABTC	Urban ABTC	Rural ABTC
**Patients given vaccine**						
Total patients given vaccine	320	47	235	130	325	47
Patients who paid for vaccine	2	2	2	0	81	0
% of need met by government	99%	96%	99%	100%	75%	100%
**Patients given eRIG**						
Total patients recommended eRIG	82	4	82	61	159	25
Total patients given eRIG	3	N/A	64	13	138	14
Patients who paid for eRIG	0	N/A	19	6	43	13
% of need met by government	4%	N/A	55%	11%	60%	4%
No. of patients who went without eRIG	79	4	18	48	21	11

However, the time period of data collection for 2017 was very short, and vaccine shortages later in the year could easily reverse the trend seen at this time point.

#### Patient expenses

Across all ABTCs, the percentage of patients arriving unaccompanied at the ABTCs was 0% for children under 15 years old, and 40.2% for those over 15. Overall, 50.1% of all patients under 15 and 43.6% of patients over 15 years were accompanied by one other person, and the remainder were accompanied by more than one person (up to a maximum of 5).

**Fig 4:**
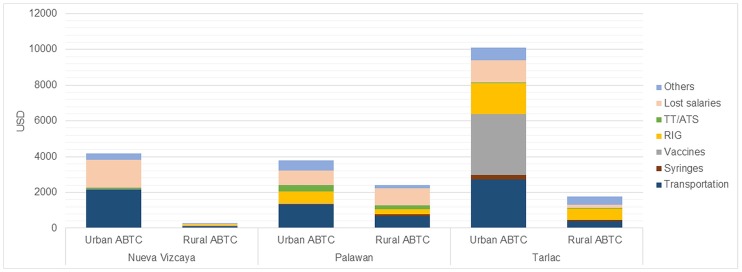
Total out-of-pocket expenses grouped by category and ABTC, 2017 in USD. TT/ATS = Tetanus Toxoid / Anti Tetanus Serum. The RIG in the rural ABTC in Nueva Vizcaya was bought by a patient and administered in an ABTC in another province.

Patients’ total out of pocket expenses (OOPE) for PEP are shown in Fig 4. Almost all of the expenses shouldered by the patients are indirect costs (transportation, lost salaries and other costs such as meals and wound care prior to going to the ABTC), but in several ABTCS patients had to contribute to the cost of RIG. Only at the urban ABTC in Tarlac did patients have to pay a considerable amount for rabies vaccine. This ABTC had the highest number of patients compared to the other 5 ABTCs in the study and suffered from considerable vaccine stock outs (shortages), forcing patients to buy vaccine in the local pharmacies at a cost two to three times that paid by the national government [5]. A detailed breakdown of OOPE by category of expense is provided in S4 Table.

The total costs were divided among the total number of patients interviewed to get the average expenditure of each patient (Table 10). Across all six clinics, the average OOPE for patients was 20.38 USD (PHP 960.48). Using data from the Department of Labor and Employment’s list of minimum wages by region, the average OOPE per visit in the 6 ABTCs (assuming 3 visits) represented 30%-160% of daily wage. The highest costs per patient were observed in both ABTCs in Tarlac. The lowest OOPE were in both of the ABTCs in Nueva Vizcaya. The OOPE at the rural Tarlac ABTC were seven times those at the rural Nueva Vizcaya ABTC, demonstrating large inequality in the cost of accessing treatment for bite wounds.

**Table 10:** Average out-of-pocket expenses (USD) per patient, 2017. Direct costs refer to those incurred for medical treatment (vaccine, RIG, tetanus immunization and consumables), indirect costs refer to travel, meals and lost salary.

ABTC	No. of Patients	Out-of-pocket Expenses (USD)			Average cost per patient
		Direct costs	Indirect costs	Total	
**Nueva Vizcaya**					
Urban ABTC	320	147.62	4021.31	4168.93	13.03
Rural ABTC	47	103.14	156.90	260.04	5.53
Pa**lawan**					
Urban ABTC	235	1341.89	2460.63	3802.53	16.18
Rural ABTC	130	708.00	1712.03	2420.03	18.62
**Tarlac**					
Urban ABTC	326	5489.33	4605.24	10094.57	30.96
Rural ABTC	47	1148.60	629.52	1778.12	37.83
**Total**	1105	8938.58	13585.63	22524.21	20.38

**Fig 5:**
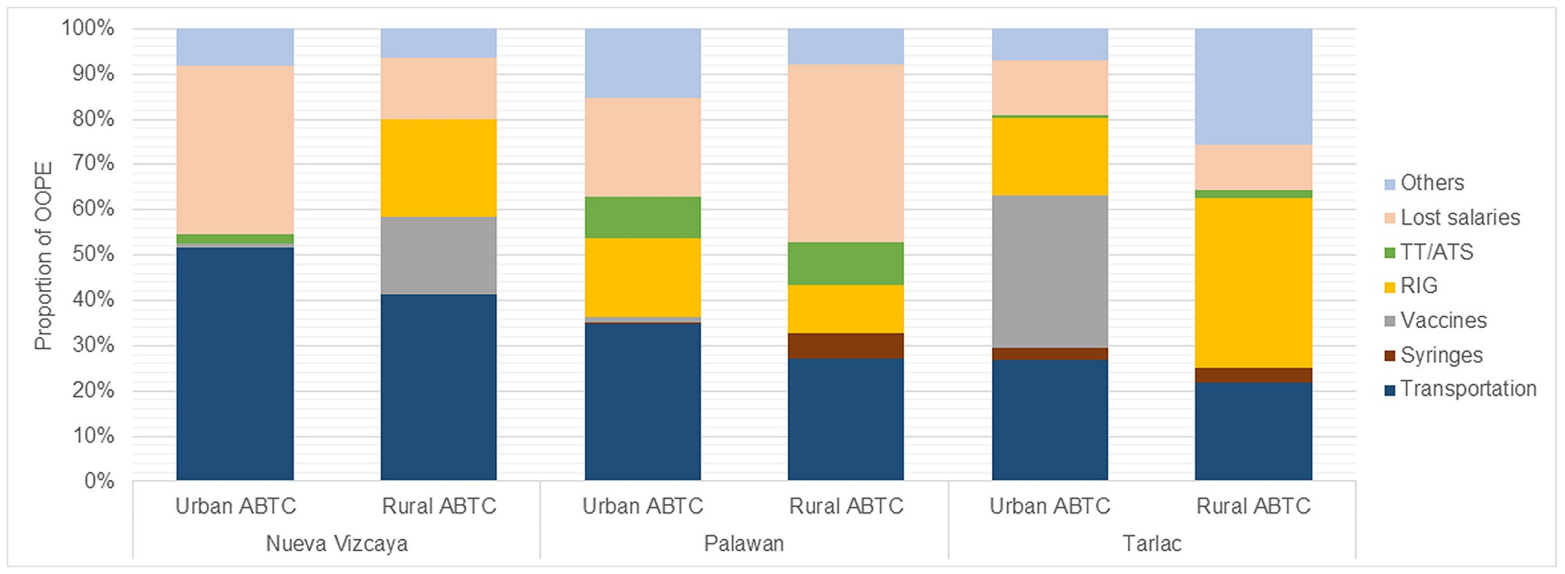
Proportion of out-of-pocket expenses by category and ABTC, 2017. TT/ATS = Tetanus Toxoid / Anti Tetanus Serum.

The proportion of out-of-pocket expenses (OOPE) related to PEP shouldered by the patients varied across all the ABTCs (Fig 5). Transportation costs comprised the majority of the OOPE in the Palawan urban ABTC and in both ABTCs in Nueva Vizcaya. While transportation was still a major expense in the other 3 ABTCs, other factors represented a significant portion of patient costs. The biggest percentage in the rural ABTC in Palawan was salaries not received by the patient and companions because of scheduled ABTC visits. Anti-rabies vaccines comprised a majority in the Tarlac urban ABTC, while RIG and other expenses such as Out-Patient Department fees made up the bulk of the total expenses in its rural counterpart.

## Discussion

The community surveys showed that bite incidences were high in both rural and urban settings, with an overall average of around 50 injuries per 1,000 population per year (5%), around a quarter of which were scratches and the rest involved bites. Overall 45% of patients sought medical treatment for their injuries, almost always in the ABTCs, and the distance to the ABTC did not seem to influence this likelihood. Of those who did not seek medical treatment, around half washed the wounds with soap and water, a third visited the traditional healer (*tandok)* and a third did nothing at all.

The community surveys showed a high animal injury incidence, but low health seeking behaviour similar to that observed elsewhere in the Philippines [8] and in other rabies endemic countries [9–13]. Awareness of where to seek treatment in the Philippines was high, but the surveys also suggested that out of pocket expenses played a role in preventing people from seeking treatment. *Tandoks* (traditional healers) were still consulted frequently for the treatment of bite wounds, but this was notably less prevalent in Palawan. In Palawan, considerable efforts have been put into community awareness of the need for PEP and training of *tandoks* to encourage them to refer patients for PEP at ABTCs. There was anecdotal evidence that a few patients were carrying out their own risk assessment of the bite and choosing not to seek treatment accordingly.

Across the cohort of 1,105 bite victims in 2017, patients incurred out of pocket expenses of between 5.53 and 37.83 USD (PHP 260.57 and 1782.55) for PEP, depending on the clinic visited and whether free government vaccine and RIG was available. Expenses incurred during each ABTC visit represented from a third up to 160% of a minimum wage earner’s daily income.

The interpretation of the data analysis assumes that the ABTCs and 30 randomly selected barangays produced representative data for each province at that these results are generally applicable to similar geographic settings in the Philippines. Vaccines are, in general, supplied to the ABTC on a quarterly basis. Since the ABTC patient survey was conducted during the first quarter of the year, interviews may have coincided with periods before these scheduled vaccine distributions. Vaccine stock outs (shortages) were identified in Tarlac that significantly affected the patients’ OOPEs.

The ABTC cohort survey showed a relatively high completion rate in 2017, but the main reason for non-completion of the PEP schedule was “no time”, likely because the patients were working, and lost salary was the 2^nd^ largest proportion of the patient out of pocket expenses.

Taken together the two surveys suggest that awareness of how to access to PEP provision is high and the travel and patient expenses do not limit patient’s access to PEP in these three provinces of the Philippines where high investment in providing PEP has been made. However, despite this, over 200 deaths from rabies a year still occur in the Philippines [5]. There is no doubt that PEP is saving thousands of lives in the Philippines each year, but before investment in opening more ABTCs is made, thorough investigation of the reasons for these remaining deaths would be helpful in determining the best course of action. In a previous study, 92% of human rabies death in the Philippines were found to be bite victims who did not seek PEP and none received timely and complete PEP [14]. There is also limited evidence of people made aware of the need for PEP and with reasonable access who still choose not to seek it and died of rabies as a result. In this context, only elimination of the disease from dogs through comprehensive vaccination campaigns will prevent human suffering and death.

Despite the relatively low treatment seeking behaviour observed at the community level, none of the households interviewed reported a death as a result of an animal injury over the past 3 years. It is likely that the most severe and risky wounds were those where treatment was sought, but of patients interviewed in the ABTCs, 85% reported that after 14 days the biting animal was alive and healthy. Other data from Philippines has indicated that only 2.2% of PEP was delivered to patients exposed to a rabid dog [15]. Information on the status of the dog 14 days after the bite proved valuable information from a health care provider’s perspective, as it lead to the vast majority of bite patients being recommended a shortened schedule. However, it is also clear that the vast majority of PEP is being used to treat injuries that were not (in retrospect) a rabies risk, and a large number were from animals known to the victim. Strengthening of dog bite prevention education strategies could reduce provoked injuries from healthy dogs and therefore the need for and cost of PEP for patients bitten. Finally there is provision in the national guidance for the Philippines that PEP can be delayed in the event of a bite from an animal with a current rabies vaccination status [16]. Good documentation of the vaccination status of animals and better coordination between animal and human health supported by this guidance could reduce PEP costs.

The surveys revealed that 5% of the population suffered a dog bite or scratch injury each year. Although awareness of local bite treatment centers was high, fewer than half of bite victims sought medical treatment, and a third sought treatment from traditional healers. Under these circumstances in a dog rabies endemic country, it is likely that costs to provide PEP will remain high and yet human deaths from rabies still continue, until effective dog vaccination programmes can eliminate the public health risk.

## Supporting information

S1 Checklist STROBE checklist. (DOC)

S1 Fig Locations of ABTCs in (A) Nueva Vizcaya, (B) Palawan, and (C) Tarlac. (TIF)

S1 Table Number of animal bites and scratches recorded by community surveys by province and by year. (DOCX)

S2 Table Additional reasons given in the community survey for not seeking medical treatment for wounds. (DOCX)

S3 Table Reasons for patients not completing the PEP series. (DOCX)

S4 Table Total Out-of-Pocket Expenses, ABTC patient survey, 2017. (DOCX)

S5 Table Biting animal status at day 28 follow-up. (DOCX)

S6 Table PEP completion status of patients bitten by animals that died or were of unknown status. (DOCX)
