# Supplementary material for: The evaluation of Animal Bite Treatment Centers in the Philippines from a patient perspective
Source: PLoS One. 2018 Jul 26;13(7):e0200873. doi: 10.1371/journal.pone.0200873 (PMC6062032; doi:10.1371/journal.pone.0200873)
Supplement: S1 Checklist — (DOC) [file pone.0200873.s001.doc]

STROBE Statement—Checklist of items that should be included in reports of ***cross-sectional studies***

|  | Item No | Recommendation |
| --- | --- | --- |
| **Title and abstract** | 1 | 1. Indicate the study’s design with a commonly used term in the title or the abstract   **Abstract, Lines 23-25 and 35-36** |
|  |  | 1. Provide in the abstract an informative and balanced summary of what was done and what was found   **Abstract, Lines 21 - 41** |
| Introduction | | |
| Background/rationale | 2 | Explain the scientific background and rationale for the investigation being reported  **Introduction, lines 47-57** |
| Objectives | 3 | State specific objectives, including any prespecified hypotheses  **Introduction, lines 78-83** |
| Methods | | |
| Study design | 4 | Present key elements of study design early in the paper  **Abstract lines 26,35**  **Introduction, line 71 - 77**  **Methods, lines 96-97** |
| Setting | 5 | Describe the setting, locations, and relevant dates, including periods of recruitment, exposure, follow-up, and data collection  **Methods, lines 96- 118 and 133-149**  **Table 1** |
| Participants | 6 | 1. Give the eligibility criteria, and the sources and methods of selection of participants   **Methods, lines 102-106 and 136-140** |
| Variables | 7 | Clearly define all outcomes, exposures, predictors, potential confounders, and effect modifiers. Give diagnostic criteria, if applicable  **Methods lines 102-113 and 143-149 and data collected are clearly described in the results section**  **No diagnostic criteria were applicable** |
| Data sources/ measurement | 8* | For each variable of interest, give sources of data and details of methods of assessment (measurement). Describe comparability of assessment methods if there is more than one group.  **Methods lines 106-113 and 143-149** |
| Bias | 9 | Describe any efforts to address potential sources of bias  **Methods lines 102-106 and 136-140** |
| Study size | 10 | Explain how the study size was arrived at  **This was based on prior experience and practicality** |
| Quantitative variables | 11 | Explain how quantitative variables were handled in the analyses. If applicable, describe which groupings were chosen and why  **Methods line 152-153 (This was largely a descriptive study)**  **Results lines 198-203, 221-223 (handling of evidence of recall bias)** |
| Statistical methods | 12 | 1. Describe all statistical methods, including those used to control for confounding   **Methods line 152-153** |
|  |  | 1. Describe any methods used to examine subgroups and interactions   **Methods line 152-153. Descriptive data for different setting provided in all results tables** |
|  |  | 1. Explain how missing data were addressed   **Methods line 106-111, line 136-140** |
|  |  | 1. If applicable, describe analytical methods taking account of sampling strategy   **Not applicable** |
|  |  | 1. Describe any sensitivity analyses   **Not applicable** |
| Results | | |
| Participants | 13* | 1. Report numbers of individuals at each stage of study—eg numbers potentially eligible, examined for eligibility, confirmed eligible, included in the study, completing follow-up, and analysed   **Results, line 166-169, 282-284** |
|  |  | 1. Give reasons for non-participation at each stage   **Not applicable** |
|  |  | 1. Consider use of a flow diagram   **Not necessary** |
| Descriptive data | 14* | 1. Give characteristics of study participants (eg demographic, clinical, social) and information on exposures and potential confounders   **Results, Table 2 and 7** |
|  |  | 1. Indicate number of participants with missing data for each variable of interest   **All relevant Results Tables** |
| Outcome data | 15* | Report numbers of outcome events or summary measures  **Results Tables 1- 10** |
| Main results | 16 | 1. Give unadjusted estimates and, if applicable, confounder-adjusted estimates and their precision (eg, 95% confidence interval). Make clear which confounders were adjusted for and why they were included   **Results Tables 1- 10** |
|  |  | 1. Report category boundaries when continuous variables were categorized   **Results Table 7 (Age range) Tables 5 and S3 (open ended question categories)** |
|  |  | 1. If relevant, consider translating estimates of relative risk into absolute risk for a meaningful time period   **Not relevant** |
| Other analyses | 17 | Report other analyses done—eg analyses of subgroups and interactions, and sensitivity analyses  **Not relevant** |
| Discussion | | |
| Key results | 18 | Summarise key results with reference to study objectives  **Discussion lines 396-414, 424-427** |
| Limitations | 19 | Discuss limitations of the study, taking into account sources of potential bias or imprecision. Discuss both direction and magnitude of any potential bias  **We consider the limitations of this type of community survey, completed in just 3 provinces and over a limited time period to be self-evident** |
| Interpretation | 20 | Give a cautious overall interpretation of results considering objectives, limitations, multiplicity of analyses, results from similar studies, and other relevant evidence  **Discussion, lines 424-456** |
| Generalisability | 21 | Discuss the generalisability (external validity) of the study results  **Discussion, Lines 402-403, 415-417** |
| Other information | | |
| Funding | 22 | Give the source of funding and the role of the funders for the present study and, if applicable, for the original study on which the present article is based  **Acknowledgments, Lines 459-463** |

*Give information separately for exposed and unexposed groups.

**Note:** An Explanation and Elaboration article discusses each checklist item and gives methodological background and published examples of transparent reporting. The STROBE checklist is best used in conjunction with this article (freely available on the Web sites of PLoS Medicine at http://www.plosmedicine.org/, Annals of Internal Medicine at http://www.annals.org/, and Epidemiology at http://www.epidem.com/). Information on the STROBE Initiative is available at www.strobe-statement.org.
