# Supplementary figures and images for: The evaluation of Animal Bite Treatment Centers in the Philippines from a patient perspective
Source: PLoS One. 2018 Jul 26;13(7):e0200873. doi: 10.1371/journal.pone.0200873 (PMC6062032; doi:10.1371/journal.pone.0200873)

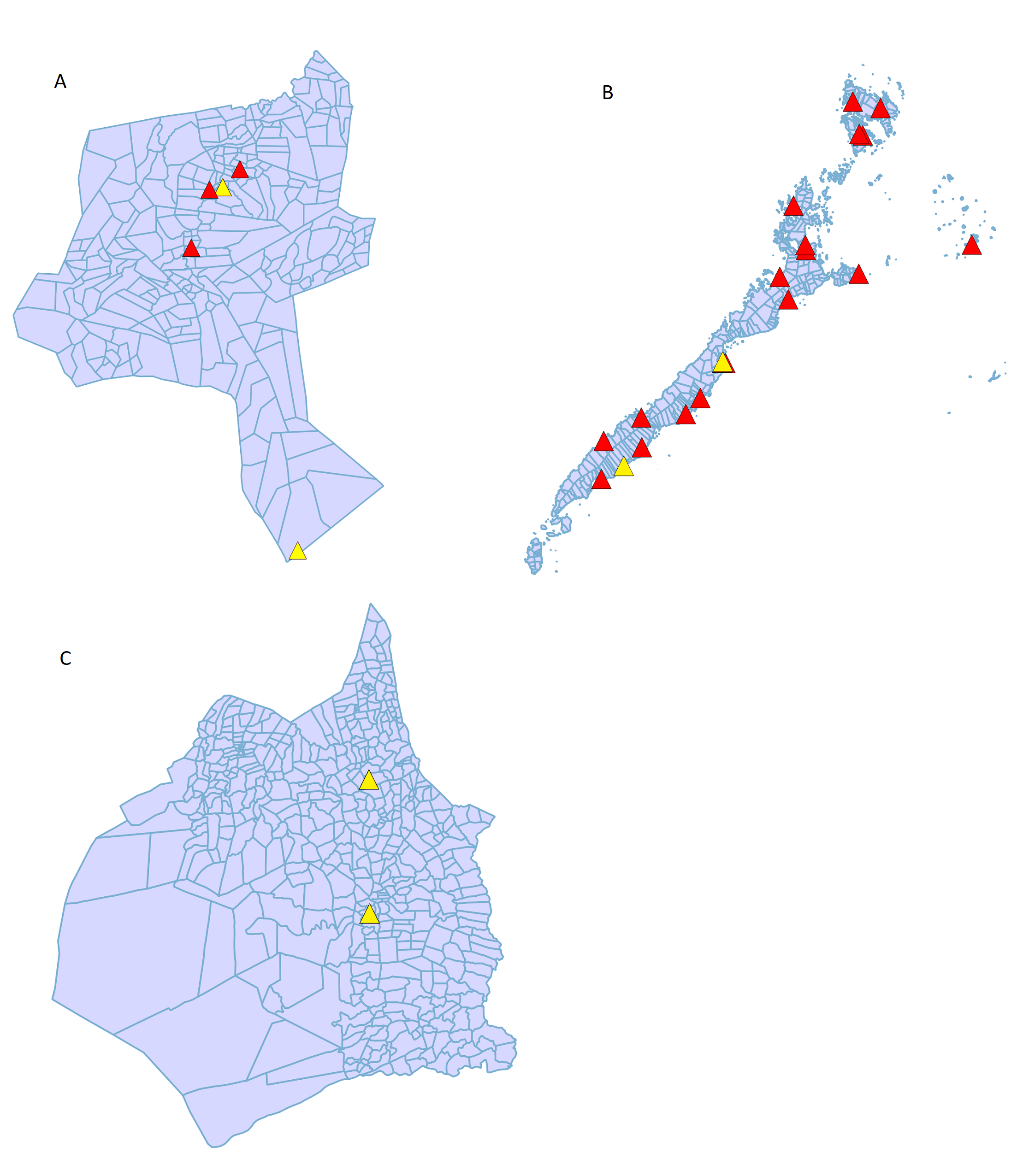

Supplement: S1 Fig — (TIF) [file pone.0200873.s002.tif]
