# Supplementary material for: The evaluation of Animal Bite Treatment Centers in the Philippines from a patient perspective
Source: PLoS One. 2018 Jul 26;13(7):e0200873. doi: 10.1371/journal.pone.0200873 (PMC6062032; doi:10.1371/journal.pone.0200873)
Supplement: S1 Table — (DOCX) [file pone.0200873.s003.docx]

| Injury type | 2014 | | | | 2015 | | | | 2016-7 | | | |
| --- | --- | --- | --- | --- | --- | --- | --- | --- | --- | --- | --- | --- |
|  | Nueva Vizcaya | Palawan | Tarlac | All | Nueva Vizcaya | Palawan | Tarlac | All | Nueva Vizcaya | Palawan | Tarlac | All |
| Bitten | 104 | 86 | 115 | 305 | 127 | 91 | 99 | 317 | 344 | 224 | 260 | 828 |
| Both | 1 | 4 | 7 | 12 | 5 | 6 | 4 | 15 | 7 | 11 | 4 | 22 |
| Scratched | 15 | 24 | 17 | 56 | 18 | 30 | 20 | 68 | 51 | 123 | 87 | 261 |
| **Total** | **120** | **114** | **139** | **373** | **150** | **127** | **123** | **400** | **402** | **358** | **351** | **1111** |
| %scratches | 12.5% | 21.1% | 12.2% | 15.0% | 12.0% | 23.6% | 16.3% | 17.0% | 12.7% | 34.4% | 24.8% | 23.5% |
