# Supplementary material for: The evaluation of Animal Bite Treatment Centers in the Philippines from a patient perspective
Source: PLoS One. 2018 Jul 26;13(7):e0200873. doi: 10.1371/journal.pone.0200873 (PMC6062032; doi:10.1371/journal.pone.0200873)
Supplement: S2 Table — (DOCX) [file pone.0200873.s004.docx]

|  | Nueva Vizcaya  (n=224) | Palawan  (n=177) | Tarlac  (n=211) | Total  (n=612) |
| --- | --- | --- | --- | --- |
| Advised not necessary | 2 | 1 |  | 3 |
| Afraid / embarrassed | 6 | 1 | 1 | 8 |
| ARV clinic moved |  | 1 |  | 1 |
| Belief in home remedies |  | 2 | 2 | 4 |
| Believed rabies is not dangerous |  |  | 1 | 1 |
| Dog was vaccinated | 2 | 5 |  | 7 |
| Didn’t know about wound at time |  | 1 | 4 | 5 |
| Doesn't like to go to ABTC | 4 |  | 1 | 5 |
| Forgot |  | 1 |  | 1 |
| No ARV in clinic |  | 3 |  | 3 |
| Too lazy |  | 1 |  | 1 |
| Victim already vaccinated | 1 | 1 | 1 | 3 |
| Decided no rabies risk | 3 | 5 |  | 8 |
| Clinic closed at the time | 1 |  | 1 | 2 |
| Worried about vaccine effect on pregnancy | 2 |  |  | 2 |
| Observed animal instead | 1 | 5 |  | 6 |
| Thought payment was needed | 2 | 1 | 1 | 4 |
