# Supplementary material for: The evaluation of Animal Bite Treatment Centers in the Philippines from a patient perspective
Source: PLoS One. 2018 Jul 26;13(7):e0200873. doi: 10.1371/journal.pone.0200873 (PMC6062032; doi:10.1371/journal.pone.0200873)
Supplement: S3 Table — (DOCX) [file pone.0200873.s005.docx]

| Reasons | Nueva Vizcaya | | Palawan | | Tarlac | |
| --- | --- | --- | --- | --- | --- | --- |
|  | Urban ABTC (n=43) | Rural ABTC (n=15) | Urban ABTC (n=72) | Rural ABTC (n=31) | Urban ABTC (n=76) | Rural ABTC (n=8) |
| Forgot schedule | 19% | 53% | 11% |  | 5% |  |
| No money | 8% | 27% | 7% | 3% | 17% | 38% |
| No time | 35% | 20% | 32% | 28% | 26% | 25% |
| Difficult to travel because of weather | 3% |  |  |  |  |  |
| Sick | 2% |  |  |  | 3% |  |
| Refused | 5% |  | 4% |  | 20% |  |
| Others | 4% |  | 3% |  | 11% |  |
| Believed it was not needed | 8% |  | 38% | 31% | 14% |  |
| No data | 3% |  | 4% | 35% | 3% | 38% |
