# Supplementary material for: The evaluation of Animal Bite Treatment Centers in the Philippines from a patient perspective
Source: PLoS One. 2018 Jul 26;13(7):e0200873. doi: 10.1371/journal.pone.0200873 (PMC6062032; doi:10.1371/journal.pone.0200873)
Supplement: S4 Table — (DOCX) [file pone.0200873.s006.docx]

| **PHP** | | Trans-portation | Meals | Medicines | Syringes | Prior tx | Vaccines | RIG | TT/ATS | Lost salaries | Others | Total |
| --- | --- | --- | --- | --- | --- | --- | --- | --- | --- | --- | --- | --- |
| Nueva Vizcaya | Urban ABTC | 101339 | 12600 | 1149 | 0 | 2024 | 2050 | 0 | 3707 | 73521 | 50 | 196440 |
|  | Rural ABTC | 5060 | 660 | 0 | 0 | 0 | 2100 | 2650 | 0 | 1673 | 110 | 12253 |
| Palawan | Urban ABTC | 62237 | 4411 | 12108 | 625 | 10155 | 2430 | 31105 | 16432 | 39142 | 530 | 179175 |
|  | Rural ABTC | 31059 | 1750 | 3417 | 6272 | 2827 | 0 | 12156 | 10676 | 45035 | 840 | 114032 |
| Tarlac | Urban ABTC | 127875 | 26950 | 2085 | 11851 | 4530 | 160791 | 81528 | 2090 | 57644 | 312 | 475656 |
|  | Rural ABTC | 18380 | 1802 | 2421 | 2521 | 1041 | 0 | 31440 | 1590 | 8440 | 16150 | 83785 |
| TOTAL |  | 345950 | 48173 | 21180 | 21269 | 20577 | 167371 | 158879 | 34495 | 225455 | 17992 | 1061341 |

| **USD** | | Trans-portation | Meals | Medicines | Syringes | Prior tx | Vaccines | RIG | TT/ATS | Lost salaries | Others | Total |
| --- | --- | --- | --- | --- | --- | --- | --- | --- | --- | --- | --- | --- |
| Nueva Vizcaya | Urban ABTC | 2150.66 | 267.40 | 24.38 | 0.00 | 42.95 | 43.51 | 0.00 | 78.67 | 1560.29 | 1.06 | 4168.93 |
|  | Rural ABTC | 107.39 | 14.01 | 0.00 | 0.00 | 0.00 | 44.57 | 56.24 | 0.00 | 35.51 | 2.33 | 260.04 |
| Palawan | Urban ABTC | 1320.82 | 93.61 | 256.96 | 13.26 | 215.51 | 51.57 | 660.12 | 348.73 | 830.69 | 11.25 | 3802.53 |
|  | Rural ABTC | 659.15 | 37.14 | 72.52 | 133.11 | 60.00 | 0.00 | 257.98 | 226.57 | 955.75 | 17.83 | 2420.03 |
| Tarlac | Urban ABTC | 2713.82 | 571.94 | 44.25 | 251.51 | 96.14 | 3412.37 | 1730.22 | 44.35 | 1223.34 | 6.62 | 10094.57 |
|  | Rural ABTC | 390.07 | 38.24 | 51.38 | 53.50 | 22.09 | 0.00 | 667.23 | 33.74 | 179.12 | 342.74 | 1778.12 |
| TOTAL |  | 7341.89 | 1022.35 | 449.49 | 451.38 | 436.69 | 3552.02 | 3371.80 | 732.07 | 4784.70 | 381.83 | 22524.21 |
