# Supplementary material for: The evaluation of Animal Bite Treatment Centers in the Philippines from a patient perspective
Source: PLoS One. 2018 Jul 26;13(7):e0200873. doi: 10.1371/journal.pone.0200873 (PMC6062032; doi:10.1371/journal.pone.0200873)
Supplement: S5 Table — (DOCX) [file pone.0200873.s007.docx]

| ABTC | | Biting animal Alive | | Dead or unknown* status of biting animal | |
| --- | --- | --- | --- | --- | --- |
| Nueva Vizcaya | Urban ABTC | 287 | 90% | 33 | 10% |
|  | Rural ABTC | 43 | 91% | 4 | 9% |
| Palawan | Urban ABTC | 192 | 82% | 43 | 18% |
|  | Rural ABTC | 105 | 81% | 25 | 19% |
| Tarlac | Urban ABTC | 271 | 83% | 55 | 17% |
|  | Rural ABTC | 41 | 87% | 6 | 13% |
| Total |  | 939 | 85% | 166 | 15% |

* Includes 43 patients lost to follow-up at Day 28
