# Supplementary material for: The evaluation of Animal Bite Treatment Centers in the Philippines from a patient perspective
Source: PLoS One. 2018 Jul 26;13(7):e0200873. doi: 10.1371/journal.pone.0200873 (PMC6062032; doi:10.1371/journal.pone.0200873)
Supplement: S6 Table — (DOCX) [file pone.0200873.s008.docx]

| ABTC | | Complete | | Incomplete | | Total |
| --- | --- | --- | --- | --- | --- | --- |
| Nueva Vizcaya | Urban ABTC | 19 | 58% | 14 | 42% | 33 |
|  | Rural ABTC | 1 | 25% | 3 | 75% | 4 |
| Palawan | Urban ABTC | 8 | 19% | 35 | 81% | 43 |
|  | Rural ABTC | 11 | 44% | 14 | 56% | 25 |
| Tarlac | Urban ABTC | 29 | 53% | 26 | 47% | 55 |
|  | Rural ABTC | 5 | 83% | 1 | 17% | 6 |
| Total |  | 73 | 44% | 93 | 56% | 166 |
